# Metabolism impacts upon *Candida* immunogenicity and pathogenicity at multiple levels

**DOI:** 10.1016/j.tim.2014.07.001

**Published:** 2014-11

**Authors:** Alistair J.P. Brown, Gordon D. Brown, Mihai G. Netea, Neil A.R. Gow

**Affiliations:** 1 Aberdeen Fungal Group, School of Medical Sciences, University of Aberdeen, Institute of Medical Sciences, Foresterhill, Aberdeen AB25 2ZD, UK; 2 Departments of Medicine, Radboud University Nijmegen Medical Center, Nijmegen and Radboud Center for Infectious Diseases, Geert Grooteplein Zuid 8, 6525 GA, Nijmegen, The Netherlands

**Keywords:** metabolic adaptation, stress adaptation, cell wall, virulence factors, regulatory networks, fungal immunology

## Abstract

Metabolism is integral to the pathogenicity of *Candida albicans*, a major fungal pathogen of humans. As well as providing the platform for nutrient assimilation and growth in diverse host niches, metabolic adaptation affects the susceptibility of *C. albicans* to host-imposed stresses and antifungal drugs, the expression of key virulence factors, and fungal vulnerability to innate immune defences. These effects, which are driven by complex regulatory networks linking metabolism, morphogenesis, stress adaptation, and cell wall remodelling, influence commensalism and infection. Therefore, current concepts of *Candida–*host interactions must be extended to include the impact of metabolic adaptation upon pathogenicity and immunogenicity.

## Highlights

*•* Metabolic adaptation impacts upon *Candida albicans* pathogenicity at multiple levels.
*•* Carbon sources influence virulence factor expression and innate immune surveillance.
*•* Nutrients also affect stress resistance and antifungal drug susceptibility.
*•* *Candida* pathogenicity and immunogenicity therefore must differ between host niches.

## Adaptation of *Candida albicans* to the host

Fungal pathogens are driven by the need to assimilate nutrients, survive, and multiply. In the short term this requires the flexibility to adapt to environmental change. In the long term this has depended on the evolution of mechanisms that permit this flexibility. The outcome for the host, although being of importance to that individual, is of secondary importance to the fungal pathogen. Following dissemination to a new host, a fungal cell attempts to assimilate local nutrients, counter any local environmental stresses, and, if possible, evade any local host defences. Recent data indicate that these adaptive processes are inextricably linked. In other words, the ability of a fungal cell to counter environmental stresses and host defences is strongly influenced by its metabolic and physiological status, and hence by local nutrient availability, reinforcing the truism ‘you are what you eat*’*. Consequently, infection outcome depends on the physiological robustness of the fungal pathogen within host niches as well as on the efficacy of host defences in these niches.

The major fungal pathogen, *Candida albicans*, is an opportunistic pathogen that is obligately associated with warm-blooded animals [1]. *C. albicans* normally thrives as a relatively harmless commensal organism in the microbiota of the skin, the oral cavity, and the gastrointestinal (GI) and urogenital tracts of most healthy individuals 1, 2. However, *C. albicans* infection can be triggered by perturbations of the normal microbiota (e.g., by antibiotic treatments), breaks in GI–blood barriers (e.g., as a result of injury or surgery), or by the use of medical implants (upon which *C. albicans* can form elaborate biofilms that seed bloodstream infection) [3]. Moreover, individuals with compromised immune defences suffer the greatest risk of *C. albicans* infection. For example, HIV/AIDS patients are highly susceptible to oral thrush, and neutropenic patients or individuals with heritable disorders in immune signalling are highly susceptible to life-threatening systemic *C. albicans* infections of the blood and internal organs 3, 4. In addition, the risk of infection is increased in diabetic patients and in those receiving parenteral nutrition [3]. In the context of this review, these observations highlight two important points. First, *C. albicans* adapts effectively to a diverse range of host niches, including nutrient availability in these niches. Second, the probability of infection is strongly influenced by the potency of the innate immune system. We argue that these factors are interrelated.

We review here current knowledge about the metabolic adaptation of *C. albicans* during commensalism and infection. We focus primarily on carbon source because of the pivotal role of central carbon metabolism, and because more is known about this aspect of metabolism. However, nitrogen, oxygen, phosphorus, sulphur, and micronutrient assimilation are also crucial for *C. albicans* pathogenicity, and many of the principles we discuss in the context of carbon are relevant to these processes. We suggest that the pivotal importance of metabolic adaptation to colonisation and disease progression extends well beyond the exploitation of available nutrients for efficient energy-generation and biomass production, and affects colonisation and disease progression at multiple levels.

## Metabolism: the platform for *C. albicans* pathogenicity

The development of powerful cellular, immunological, molecular, and genomic tools has empowered rapid advances in our understanding of *C. albicans* pathobiology, elevating this fungus to the status of a model fungal pathogen. For some time it has been clear that a defined set of virulence factors promote *C. albicans* pathogenicity, including yeast–hypha morphogenesis, phenotypic switching, adhesins, invasins, and secreted hydrolases 1, 2. More recently the application of unbiased genome-wide screens has reminded us that multifarious fitness attributes are also crucial to *C. albicans* pathogenicity. These include the metabolic capacity to assimilate the host nutrients that support cell division, the resistance to physiologically relevant stresses imposed in host microenvironments, the tolerance to the elevated temperatures of the host, and the construction of a robust cell wall 5, 6, 7, 8, 9. Metabolism provides the platform upon which all other fitness attributes depend, generating the precursors and energy required for cell wall biosynthesis, antioxidant production, macromolecular repair, and protein refolding, for example. The essentiality of metabolism means that fungal specific pathways, or key enzymes with fungal specific catalytic mechanisms, represent potential targets for antifungal drug therapies 10, 11.

*C. albicans* cells display efficient metabolic adaptation to host microenvironments, rapidly tuning their metabolism to the available nutrients. These microenvironments are complex, dynamic, and often glucose-limited. For example, glucose levels are maintained at around 0.06–0.1% (3–5 mM) in the bloodstream, and are around 0.5% in vaginal secretions 12, 13. Consequently, the expression of key metabolic functions is controlled in a niche-specific fashion during host colonisation, commensalism, and disease progression 12, 14, 15 (Table 1). *C. albicans* cells induce glycolytic, tricarboxylic acid cycle, and fatty acid β-oxidation genes during mucosal invasion 16, 17. In the bloodstream and during renal infection, *C. albicans* populations are heterogeneous, individual cells displaying glycolytic activity (hexose catabolism) or gluconeogenic activity (hexose anabolism), depending upon their immediate microenvironments 12, 18, 19. Following phagocytosis by macrophages and neutrophils, *C. albicans* cells display expression patterns that reflect carbon starvation, activating enzymes involved in fatty acid β-oxidation, the glyoxylate cycle, and gluconeogenesis 19, 20, 21. Lactic acid metabolism is essential for GI colonisation [22], and this non-fermentable carboxylic acid is present at significant concentrations in the vagina (∼0.4%: 45 mM) [13]. Therefore, *C. albicans* cells thrive in host microenvironments that contain contrasting carbon sources.

**Table 1:** *Candida albicans* carbon metabolism in host niches

	Gene regulation^a^				
Host niche	Glycolysis	Gluconeogenesis	Glyoxylate cycle	Fatty acid β-oxidation	Refs
Blood plasma					[19]
Neutrophils					12, 17, 19, 21, 56; Mette Jacobsen, PhD thesis, Aberdeen University, 2005
Macrophages					12, 20; Mette Jacobsen, PhD thesis, Aberdeen University, 2005
Oral mucosa					16, 17
Kidney^b^	/	/	/	/	[12], Mette Jacobsen, PhD thesis, Aberdeen University, 2005
Liver^c^					17, 58

a Upregulation (red arrows), downregulation (blue arrows), and no significant regulation (grey arrows) are expressed relative to the control *C. albicans* cells used in each transcript profiling experiment. Upregulation or downregulation is inferred on the availability of data for some (not all) of the genes on these pathways. These expression patterns display temporal regulation.

b Population heterogeneity in the expression patterns is observed by single cell profiling, presumably because of variability in the availability of host carbon sources between immediate cellular microenvironments and the local consumption of these carbon sources by the invading fungus.

c The upregulation of genes involved in both hexose catabolism and anabolism in these transcript profiling experiments could be due to the population heterogeneity of *C. albicans* cells colonising the liver.

Metabolic adaptation is controlled by complex transcriptional networks in *C. albicans* 9, 23, 24. The cellular roles of some of these networks have been conserved during yeast evolution, such as the general control of amino acid metabolism (GCN response) 9, 25 and sugar-sensing pathways [26]. Interestingly, the regulation of central carbon metabolism has undergone major transcriptional rewiring in *C. albicans* relative to *Saccharomyces cerevisiae*. For example, glycolysis is induced by Gcr1 in *S. cerevisiae*, but by Gal4 and Tye7 in *C. albicans* 9, 23, 24.

The importance of metabolic adaptation for GI colonisation and systemic infection has been highlighted by the elaboration of regulatory networks that are required for these processes in *C. albicans*. *In vivo* genetic screens have indicated that Tye7 (a glycolytic activator) is specifically required for GI colonisation, whereas Rtg1/3 and Hms1 (which modulate hexose catabolism) promote both GI colonisation and systemic infection [27]. Metabolic adaptation within host niches is linked in part to the morphological states of the fungus in these niches, such as yeast, pseudohyphal, and hyphal cells, white and opaque cells, and the recently described GUT (‘Gastrointestinally-indUced Transition’) phenotype 28, 29, 30. Nevertheless, metabolic adaptation is integral to *C. albicans* commensalism and pathogenicity.

## Carbon adaptation modulates stress resistance

Metabolism also promotes the virulence of *C. albicans* indirectly by enhancing stress adaptation. Stress resistance is required for *C. albicans* virulence: it increases the survival of fungal cells in host niches by reducing their vulnerability to local environmental stresses and to phagocytic killing 5, 31, 32. Metabolism contributes to stress adaptation by generating molecules such as the osmolyte glycerol, antioxidants such as glutathione, and the stress protectant trehalose [5]. Therefore, the ability of *C. albicans* cells to respond to environmental stress is likely to depend upon the preadapted metabolic state of these cells, and hence upon available nutrients in host microenvironments. Almost without exception, however, the analysis of *C. albicans* stress responses has been performed on cells cultured on rich, glucose-containing media [5] that differ significantly from host microenvironments which are often glucose-limited (above). Significantly, recent data indicate that changes in carbon source exert dramatic effects upon the stress resistance of *C. albicans* 33, 34.

Transient exposure to glucose induces the transcription of *C. albicans* genes involved in oxidative stress adaptation, thereby enhancing cellular resistance to acute oxidative stress [34]. This phenomenon, which is regulated by glucose-sensing pathways, probably reflects adaptive prediction [35] whereby *C. albicans* has ‘learnt’ over evolutionary time to anticipate phagocytic attack following entry to the bloodstream [8]. Interestingly, the contrasting evolutionary pressures experienced by *C. albicans* and its relatively benign cousin *S. cerevisiae* have yielded contrasting predictive stress responses to glucose. Glucose enhances oxidative stress resistance in *C. albicans*, whereas it decreases stress resistance in *S. cerevisiae* via protein kinase A (PKA)-mediated repression of the core transcriptional response to stress 36, 37. The differing effects of glucose in these yeasts are largely due to the functional rewiring of Msn2/4 transcription factors, which activate this core stress response in *S. cerevisiae* and *Candida glabrata* 37, 38, but control weak acid stress responses in *C. albicans* [39].

Carbon adaptation also affects resistance to osmotic stress and antifungal drugs 33, 40. These effects, which are mediated partly through PKA signalling [41], may relate to the effects of metabolic adaptation upon the cellular abundances of osmolytes such as glycerol, and antioxidants such as glutathione and trehalose 34, 42, 43. Glycerol and trehalose are synthesised via short metabolic branches off the glycolytic pathway. However, these effects also involve *C. albicans* cell wall remodelling 33, 40. Growth on different carbon sources yields *C. albicans* cells with cell walls that differ architecturally and biophysically (below). These preadapted cell walls confer cells with differing abilities to survive the imposition of osmotic and cell wall stresses. For example, compared with cells grown on glucose, lactate-grown cells display increased resistance to osmotic stress, amphotericin B, and caspofungin, and reduced resistance to an azole antifungal drug [33]. These alterations, which have been observed for a range of carbon sources including other sugars (fructose and galactose), other carboxylic acids (pyruvate), lipids, and amino acids [33], correlate with carbon source-mediated changes in the cell wall proteome [40]. These studies suggest that the metabolic adaptation of *C. albicans* cells to local nutrients within host niches influences their ability to rebut local environmental stresses and resist antifungal drug therapy.

## Carbon adaptation triggers cell wall remodelling

**Figure 1:**
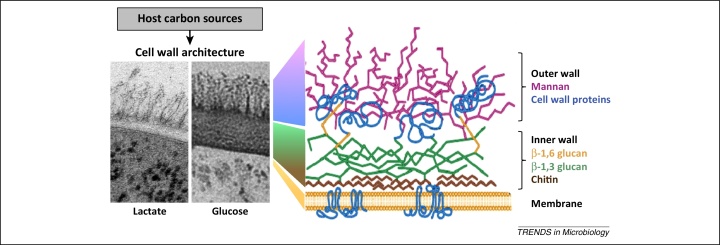
Changes in carbon source programme major changes in cell wall architecture. Transmission electron micrographs of the *Candida albicans* cell wall from cells grown on lactate or glucose as sole carbon source are shown on the left [33]. The cartoon on the right illustrates the structure of the *C. albicans* cell wall (adapted, with permission, from [76]).

Changes in carbon source exert dramatic effects on the *C. albicans* cell wall [33]. The cell wall has a characteristic architecture comprising a relatively thick inner lattice of β-glucan and chitin that is decorated with a dense coat of mannan fibrils (Figure 1) 6, 44. These mannans represent heavily *N-* and *O-*glycosylated proteins, many of which are covalently crosslinked to the carbohydrate infrastructure 45, 46. This structure has been defined for *C. albicans* cells grown on glucose. The relative proportions of β-glucan, chitin, and mannan are similar for glucose- and lactate-grown cells. However, cell wall biomass is significantly reduced after growth on lactate, and the cell walls of lactate-grown cells possess a β-glucan and chitin layer that is half the thickness of that for glucose-grown cells (Figure 1) [33]. The dynamics of these changes, which have not been described, probably depend upon the construction of new cell wall. Furthermore, significant changes were observed in the cell wall proteome [40]. Significantly, the levels of many cell wall remodelling enzymes were affected by carbon source, including glucanosyltransferases (Pga4, Phr1, and Phr2), glucosyltransferases (Bgl1), and transglycosylases (Crh11). Therefore, changes in crosslinking between cell wall biopolymers no doubt contribute to the altered biophysical properties of lactate-grown cell walls, which are more porous, more hydrophobic, and less elastic than those of glucose-grown cells [33].

The mechanisms by which carbon source influences *C. albicans* cell wall structure remain obscure, but are likely to involve a combination of metabolism and signalling. Numerous links probably exist between the complex regulatory networks that control sugar sensing (which include AMP kinase and adenylyl cyclase–PKA signalling) and cell wall biosynthesis and remodelling in *C. albicans* (which involve PKC, calcineurin, and stress signalling) 6, 26. In addition, carbon fluxes into cell wall macromolecules must differ significantly during growth on sugars and on secondary carbon sources such as lactate because the generation of hexoses required for β-glucan, mannan, and chitin synthesis via gluconeogenesis is an energy-demanding process. Irrespective of the mechanisms by which they occur, the effects of carbon source upon cell wall architecture that have been defined *in vitro* are likely to be highly relevant to host niches because changes in cell wall structure take place following cultivation in blood and under vaginal simulating conditions 47, 48. Therefore, local carbon sources in host niches must have a strong influence on the architecture and functionality of the *C. albicans* cell wall.

## Metabolic adaptation influences virulence factors

**Figure 2:**
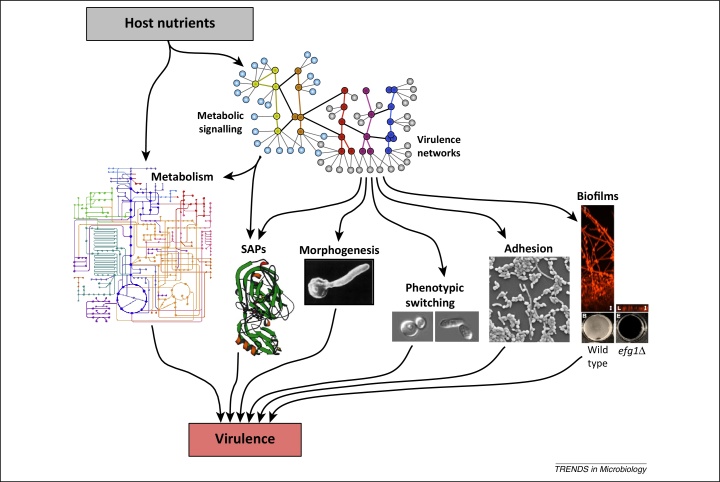
Nutrients influence the expression of key virulence factors in *Candida albicans*. Nutrient adaptation influences the expression of secreted aspartic proteases (SAPs), yeast–hypha morphogenesis, adhesion, and biofilm formation via an integrated network of metabolic and virulence signalling pathways. The images of white and opaque cells are from Zordan *et al.*[61], and the image of *C. albicans* adhesion is from Delgado-Silva *et al.*[100]. The SAP crystal structure is reproduced, with permission, from Cutfield *et al.*[101], and the biofilm images from Nobile *et al.*[72].

In addition to enhancing stress resistance and promoting cell wall remodelling, there are longstanding reports showing that metabolic adaptation influences the pathogenicity of *C. albicans* by modulating the expression of key virulence factors (Figure 2). For example, glucose is one of several stimuli that can trigger hyphal morphogenesis, and glycolytic genes are induced during this yeast-to-hypha transition 49, 50. In addition, metabolic genes account for approximately one-third of genes that are regulated during the white–opaque phenotypic switch: white cells upregulate glycolytic genes whereas opaque cells upregulate genes involved in respiratory metabolism [29]. The expression of secreted aspartic proteinase (SAP) genes is regulated in response to available nitrogen and carbon sources [51]. For example, *SAP2* is expressed at high levels during growth on glycerol, at medium levels on glucose or galactose, and at low levels when *C. albicans* cells are grown on ethanol [51]. Furthermore, growth on different dietary sugars strongly influences the adhesion of *C. albicans* to abiotic and host surfaces 52, 53, and also biofilm formation [54].

Therefore it is not surprising that key metabolic functions are regulated alongside other fitness attributes and virulence factors in a niche-specific fashion during host colonisation, commensalism, and disease progression 14, 15. For example, following exposure to macrophages or neutrophils, *C. albicans* induces genes required for the assimilation of secondary carbon sources and amino acid biosynthesis alongside genes involved in oxidative stress adaptation (*SOD1, CAT1*, and *GPA3*), hyphal growth (*ECE1*), and adhesion (*HWP1*) 12, 20, 21, 55, 56. This coregulation of fitness and virulence is also observed during GI colonisation, mucosal invasion, and systemic infection 16, 17, 28, 57, 58.

Metabolic adaptation appears to be coordinated actively with the regulation of key virulence factors via complex signalling networks. For example, the adenylyl cyclase–PKA–Efg1 signalling axis controls carbon metabolism (in part via Tye7) as well as yeast–hypha morphogenesis, white–opaque phenotypic switching, and stress resistance 41, 59, 60, 61, 62. The TOR (‘target of rapamycin’)–Nrg1 and Gcn2–Gcn4 signalling axes regulate metabolism as well as filamentous growth 8, 9, 25, 63, 64, 65. These interconnected regulatory networks contribute differentially to host colonisation and infection because the inactivation of Efg1 attenuates systemic infection but promotes GI colonisation 57, 66. The view that metabolism actively modulates other *C. albicans* virulence attributes is reinforced by at least two additional observations. First, mutations that disrupt key aspects of metabolism also affect cell wall integrity, stress sensitivity, virulence factors, and pathogenicity 12, 28, 67, 68, 69, 70, 71, 72. Second, regulatory networks that are required for GI colonisation or systemic infection include metabolic components [27].

The impact of metabolism upon the expression of virulence factors can be indirect, involving longer-term temporal relationships that highlight the dynamic nature of local niches. The stimulation of yeast-to-hypha morphogenesis via amino acid catabolism provides an excellent example of this [73]. In the absence of glucose, *C. albicans* cells exploit amino acids as a carbon source, excreting the excess nitrogen in the form of ammonia. This raises the ambient pH of the local environment, thereby triggering hyphal development [73]. It is conceivable that this phenomenon might help to protect *C. albicans* cells against macrophage killing by inhibiting acidification of the phagolysosome, as well as promoting morphogenesis in other host niches [74].

## Carbon adaptation modulates immune surveillance

Metabolic adaptation also influences *C. albicans* pathogenicity at a further level – by modulating immune surveillance. Phagocytic cells (primarily macrophages and neutrophils) play a key role in preventing *C. albicans* infection [75]. These innate immune cells attempt to recognise *C. albicans* cells initially via pattern recognition receptors (PRRs) that detect specific pathogen-associated molecular patterns (PAMPs) on the fungal cell surface. Fungal β-glucan is detected through Dectin-1, and mannans via TLR4, Dectin-2, DC-SIGN, MINCLE, and macrophage mannose receptor 1 75, 76. These initial PAMP–PRR interactions activate phagocyte intracellular signalling pathways, for example via the Dectin-1/SYK/CARD9 and TLR4/TRIF–MYD88 pathways. This triggers induction of antimicrobial effector mechanisms such as the respiratory burst, as well as the release of a variety of proinflammatory cytokines, chemokines, and lipids that stimulate other leukocytes and attract them to the site of infection [75].

Following phagocytosis, a skirmish ensues between the *C. albicans* cell and the phagocyte. The fungal cell is trapped within the phagosome, which then undergoes maturation and lysosomal fusion to create the phagolysosome [77]. During this process the phagocyte attempts to kill the fungal cell by exposing it to a combination of reactive oxygen and nitrogen species, protease activation, potassium fluxes, and decreased ambient pH 75, 78, 79. Meanwhile, *C. albicans* attempts to respond by activating oxidative stress responses and by forming hyphae that are capable of rupturing and killing the phagocyte 78, 80, 81.

The outcome of the battle between fungus and host, which depends upon numerous individual skirmishes, has a major impact upon disease outcome. In general, in healthy individuals invading *C. albicans* cells are cleared effectively by innate immune defences. By contrast, the balance can be tipped towards the development of potentially lethal systemic infections by factors that attenuate phagocytic potency (such as immunosuppression or heritable defects in phagocytic signalling pathways [75]) or that promote fungal colonisation (such as antibacterial therapies or biofilm formation on implanted medical devices [2]). Therefore, growth conditions that increase the physiological robustness of the fungus may tip the balance, reducing phagocytic efficacy.

**Figure 3:**
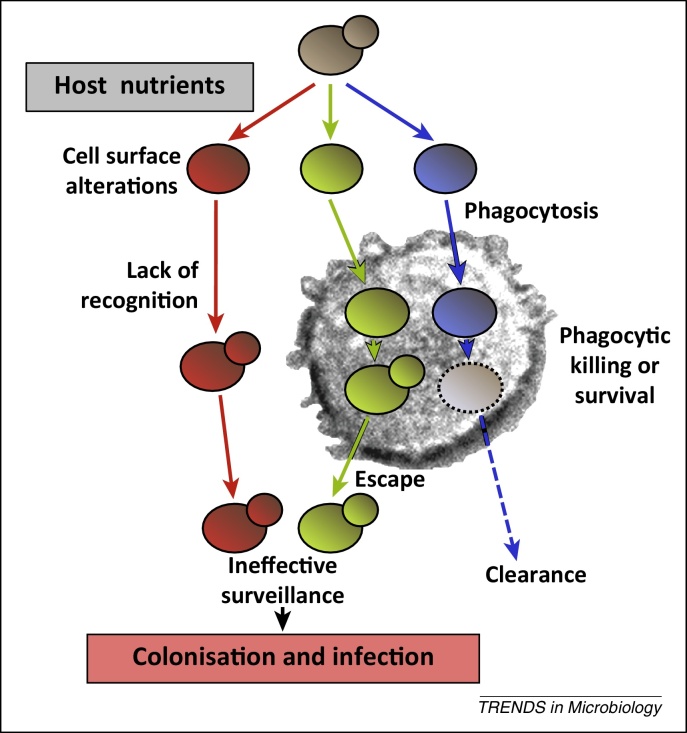
Changes in carbon source impact on immune surveillance by altering the recognition of *Candida albicans* cells by innate immune cells and by reducing the susceptibility of the fungal cells to phagocytic killing via elevated oxidative stress resistance [8].

The metabolic environment at the site of infection affects the interaction between the fungus and the immune cells. *C. albicans* cells grown on lactate are less visible to the immune system than cells grown on glucose (Figure 3) [82]. Compared with glucose-grown cells, lactate-grown *C. albicans* cells stimulate the production of more interleukin-10 (IL-10) and less IL-17 by human peripheral blood mononuclear cells from healthy volunteers. This trend is observed for a range of clinical *C. albicans* isolates from different epidemiological clades and from different host niches [82]. Lactate-grown *C. albicans* cells are also phagocytosed less efficiently by murine macrophages. Furthermore, those lactate-grown *C. albicans* cells that are engulfed by the macrophages are better able to kill and escape from macrophages (Figure 3). These observations suggest that growth on a non-fermentable secondary carbon source renders *C. albicans* cells less visible to the immune system, and less easy to kill than glucose-grown cells. Therefore, the mechanistic basis for the reduced immune visibility of lactate-grown *C. albicans* cells probably lies in the cell wall because many of the key PAMPs involved in immune recognition are located in the cell wall, and changes in cell wall structural components are known to affect immune recognition 48, 81, 83, 84, 85. However, these changes in immune visibility do not simply relate to the altered fluxes to cell wall biosynthesis during growth on glucose or lactate because *C. albicans* cells grown on a mixture of glucose plus lactate possess thick cell walls [33] but even so invoke similar immune responses to lactate-grown cells [82].

Additional observations link metabolic adaptation with cell wall structure and immune recognition. For example, *C. albicans* opaque cells differ from white cells with respect to their metabolism, cell surface properties, and immunogenicity [86]. Furthermore, these properties are mechanistically linked by well-defined mutations that affect white–opaque switching, metabolism, and host interactions 59, 61, 62. *C. albicans* scavenges the micronutrients zinc via the pH-regulated antigen Pra1 [87] and ferritin iron via the hypha-specific cell wall adhesin Als3 [88]. Hyphal development and hypoxia involve metabolic adaptation, changes to the cell wall, and changes in immune recognition 47, 76, 81. Furthermore, differences in the Dectin-1-mediated clearance of *C. albicans* cells during systemic infection have been shown to relate to the differential activation of cell wall biosynthetic functions *in vivo* within renal microenvironments [89].

Recent data indicate that local microenvironments within host niches also affect the metabolism and functionality of innate immune cells, and subsequently their interaction with *C. albicans*. A crucial role was recently proposed for glucose metabolism in the activation of immune cells. Whereas naïve and resting cells metabolise glucose mainly via oxidative phosphorylation, a switch to aerobic glycolysis (also termed the ‘Warburg effect’) is crucial for proliferating lymphocytes [90], and subsequently for important anti-*Candida* mechanisms such as the deployment of a T helper 17 (Th17) response [91]. Moreover, recent studies have shown that glycolysis and succinate play key roles in modulating the capacity of the innate immune system to mount a proper inflammatory response [92]. The important impact of glucose metabolism upon immune function leads to the hypothesis that it also modulates antifungal host defence. This hypothesis, which needs further study, has been recently strengthened by the observation that in epithelial cells mTOR (a key regulator that integrates nutrient inputs and energy levels to control cell growth and proliferation) is central for protection against *C. albicans*-induced cell damage [93]. Another example of metabolic modulation of immune responses is provided by endogenous tryptophan catabolism in the GI mucosa, which promotes IL-22 production by innate lymphoid cells, which in turn enhances intestinal immunity and protection against *C. albicans* [94]. Therefore, the local metabolic environment of the host contributes, together with the metabolic adaptation of *C. albicans*, to the efficacy or failure of local immune surveillance mechanisms.

## Concluding remarks and future perspectives

**Figure 4:**
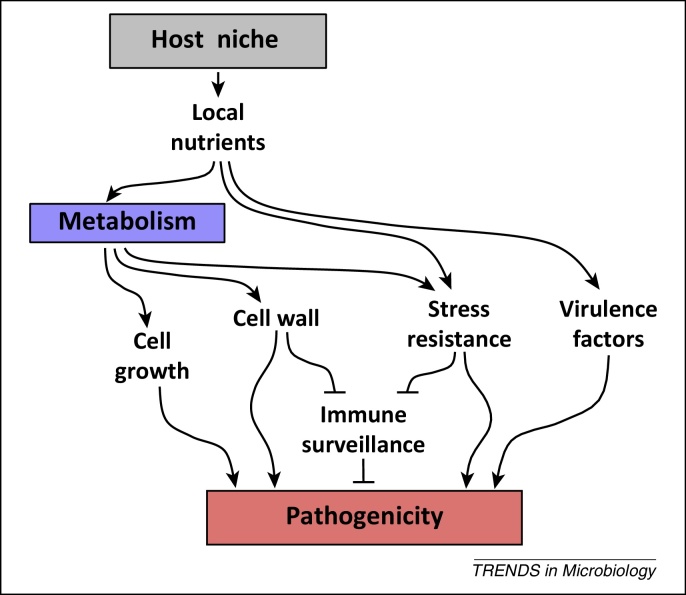
Nutrient adaptation affects *Candida albicans* pathogenicity at multiple levels. Nutrient adaptation contributes directly to pathogenicity by supporting fungal growth. Nutrient adaptation also promotes pathogenicity indirectly through cell wall remodelling, by enhancing stress resistance, by modulating the expression of key virulence factors, and by affecting the efficacy of immune surveillance by innate immune cells (see text).

To summarise, metabolic adaptation impacts upon *C. albicans* pathogenicity at multiple levels: by promoting nutrient assimilation, cell wall remodelling, stress resistance, and the expression of virulence factors, and also by influencing immune surveillance (Figure 4). In addition, metabolic adaptation affects antifungal drug susceptibility 33, 40. Consequently, preadaptation to different growth conditions affects the virulence of *C. albicans* cells during systemic and mucosal infection 33, 95, as well as the ability to clear these infections with antifungal drugs [96]. We reason, therefore, that during infection *C. albicans* cells growing in host niches with differing nutrient availabilities will display differing degrees of stress resistance and virulence, will vary with respect to their vulnerability to innate immune defences, and will differ with respect to their susceptibility to antifungal therapy.

Numerous challenges lie ahead (Box 1). It will be important to define the mechanistic links that connect metabolic adaptation with stress resistance, with drug susceptibility, and with innate immune recognition and killing. As well as being important for our understanding of *C. albicans* pathobiology, these mechanistic links could conceivably provide therapeutic approaches that might augment the activities of current antifungal drugs. For example, compounds that block nutrient-enhanced antifungal drug resistance, glucose-enhanced oxidative stress resistance, or carbon-induced cell wall remodelling would increase the susceptibility of *C. albicans* to antifungal therapy and/or host immune defences. In this respect it is important to note that rapamycin, as an inhibitor of TOR and glucose-sensing, was initially developed as a novel antimycotic drug [97]. Unfortunately, its immunosuppressive effects (through the concomitant inhibition of mTOR in immune cells), has precluded its full development for treatment of human fungal diseases. Identification of specific inhibitors for the fungal TOR has the potential to represent an important therapeutic avenue.

Box 1 **Outstanding questions**

• What molecular mechanisms interconnect metabolic adaptation with stress resistance, cell wall remodelling, and antifungal drug susceptibility in *Candida albicans*?
• Are there points of fragility within these regulatory networks that can be exploited pharmaceutically to augment current antifungal therapies?
• Which nutrient-dependent changes in the molecular architecture of the *C. albicans* cell wall moderate recognition by innate immune cells?
• Which host nutrients (in addition to carbon source) exert the greatest effects upon the ability of innate immune cells to recognise and kill *C. albicans*?
• How does local nutrient adaptation within divergent niches affect fungal fitness and immune surveillance during host colonisation and infection?
• Can these regulatory mechanisms be exploited to enhance immune surveillance, promote phagocytic killing, and reduce infection?
• How relevant are the effects of metabolic adaptation upon virulence to other major fungal pathogens of humans such as *Cryptococcus neoformans*, *Aspergillus fumigatus, Pneumocystis*, and the dimorphic fungal pathogens?
• To what extent have the relevant regulatory networks in these pathogens been evolutionarily tailored to their host niches, given that some of these fungi also occupy environmental niches?

It will be particularly challenging to characterise the links between metabolism, stress and drug resistance, virulence, and immune surveillance in the context of infection. Host niches are complex and dynamic. Local nutrient availability changes constantly during colonisation and disease progression as the fungus assimilates host nutrients, and the host responds to the fungus [98]. Individual host niches display considerable spatial as well as temporal heterogeneity which, in combination with the stochasticity of gene expression, contribute to the population heterogeneity of *C. albicans* cells within host niches 12, 55. Therefore, in addition to powerful genome-wide screens and expression profiling 16, 17, 27, 57, 58, 71, 72, new experimental approaches must be exploited to define the spatial and temporal behaviours of individual cells and lesions within infected tissues. These might involve, for example, 2D elemental, molecular, and luminescent imaging of lesions and tissues during infection with well-defined *C. albicans* strains and mutants 12, 55, 98, 99. When combined with the local dissection of immunological responses to fungal infection, these approaches will provide invaluable insights into fungus–host interactions during colonisation and infection. Meanwhile, we argue that current perceptions of *Candida–*host interactions must incorporate the influences of metabolic adaptation upon pathogenicity and immunogenicity.
